# Working in corona-designated departments in a fortified underground hospital: Concerns about corona and predictors of job burnout

**DOI:** 10.3389/fpsyt.2023.1105632

**Published:** 2023-03-07

**Authors:** Lauren Nashashibi, Marlyn Khouri, Irit Meretyk, Tom Livni, Noga Cohen, Eyal Fruchter

**Affiliations:** ^1^Rambam Health Care Campus and Faculty of Medicine, Technion – Israel Institute of Technology, Haifa, Israel; ^2^Department of Special Education, University of Haifa, Haifa, Israel; ^3^The Edmond J. Safra Brain Research Center for the Study of Learning Disabilities, University of Haifa, Haifa, Israel

**Keywords:** COVID-19, job burnout, physicians, corona-designated departments, health care, healthcare profession, psychiatry, psychology

## Abstract

**Background:**

In August 2020 during Israel’s second COVID-19 wave Rambam Medical Center opened the Sammy Ofer Fortified Underground Emergency Hospital. This was declared a regional Corona center in the north of Israel, receiving the most severe Corona patients from the region. Alongside the advanced inpatient capacity and technology within the underground facility, there was a severe shortage of trained medical and paramedical staff, as well as harsh working conditions. The current study examined the implications and effects of working in an underground facility on healthcare workers, focusing on emotion regulation tendencies and profession as predictors of job burnout.

**Methods:**

Seventy-six healthcare workers, who had worked in the underground hospital for a minimum continuous period of 2 weeks during the peak of the COVID-19 pandemic, and a control group of 40 healthcare workers from northern Israel were asked to fill out an online survey administered *via* Qualtrics (total sample 116). The survey comprised six questionnaires: a demographic survey questionnaire; a COVID-19 concerns questionnaire; a psychological distress questionnaire (DASS, Depression Anxiety Stress Scale); trait worry (PSWQ; Penn State Worry Questionnaire); emotion regulation (ERQ, Emotion Regulation Questionnaire), and burnout (SMBM, Shirom - Melamed Burnout Measure).

**Results:**

Independent-samples *t*-tests revealed no significant differences in psychological distress or burnout between Rambam Underground hospital workers and the control group. Conversely, COVID-19 concern scores were significantly different in the two groups, the Rambam hospital workers showing less concern (*M* = 2.9, *SD* = 0.73) than the control group (*M* = 3.47, *SD* = 0.76) [*t*_(114)_ = −3.974, *p* < 0.001]. Hierarchical linear regression analysis identified the significant predictors of burnout among healthcare workers. Participants’ profession (physician), psychological distress (total DASS score), and a personality trait of worry were statistically significant predictors for job burnout (*p* = 0.028, *p* < 0.001, *p* = 0.023, respectively). Concerns about COVID-19 marginally predicted job burnout (*p* = 0.09). Group (underground vs. control) and emotion regulation tendencies did not predict burnout.

**Conclusion:**

The two groups showed no significant differences in psychological distress nor in burnout. Being a physician, having an intrinsic trait of being overly worried and experiencing psychological distress were significant predictors for job burnout among healthcare workers, regardless of work environment (underground vs. control).

## Introduction

In December 2019 the corona virus (COVID-19) began spreading around the world from Wuhan, China. The clinical manifestations of COVID-19 infection are those of a respiratory disease, whose severity depends in part on the patient’s age, medical history, and general physical condition. In previously healthy individuals the infection will mostly cause only mild symptoms, while older and/or sicker individuals are prone to develop severe disease, possibly resulting in respiratory failure and even death (1). During Israel’s second COVID-19 wave in August 2020 289,799 people were infected, of whom 845 were critically ill, 232 of them requiring a ventilator. According to data from the Ministry of Health, the daily number of infected individuals in this period ranged from 3,708 to 5,691 despite a strict quarantine policy (2). These unique and rather extreme circumstances, forecasting a steep increase in infections rate and morbidity, led to Rambam Medical Center’s decision to open the Sammy Ofer Fortified Underground Emergency Hospital.

The Sammy Ofer underground hospital was built to accommodate and maintain the full range of Haifa’s clinical activity under all possible conditions of external threat, for example, in times of war and pandemic. It was established after the attack on Haifa’s hospitals during Israel’s Second Lebanon War and is considered the largest of its kind in the world. It covers an area of 60,000 square meters, has capacity for 2,000 beds, and contains operating rooms as well as other complex treatment infrastructure (3).

When it opened due to COVID-19, the hospital was declared a regional Corona center in the north of Israel, with 770 dedicated beds and 170 respirators for receiving the most severe Corona patients. One of the biggest shortcomings during this period was that, alongside the advanced inpatient capacity and technology within the underground facility, there was a severe shortage of trained medical and paramedical staff. These personnel had to work in an infected facility with the risk of bringing the potentially lethal infection back to their family members.

Several studies published during the past years have shown that healthcare workers who treated COVID-19 patients suffered more frequently from psychological distress and mental health issues, such as depression and anxiety, than those who did not treat COVID-19 patients (4–6). Psychological distress is defined as emotional suffering accompanied by symptoms of depression and anxiety (7). The distress can be triggered by personality traits and maladaptive coping strategies such as the tendency to worry or become concerned, i.e., a chain of relatively uncontrollable thoughts attempting to find solutions for issues with uncertain results (8, 9). Worrying may also be characterized by the inability to let go of negative emotions leading to anxiety, stress and eventually depression (10, 11).

Mental and psychological distress may also be associated with burnout at the workplace (12), particularly among populations experiencing prolonged work-related stress (13). Burnout syndrome refers to emotional exhaustion occurring among workers, regardless of a particular profession (14). An Israeli study in 2006 proposed a multidimensional approach to burnout syndrome, suggesting that it derives from emotional exhaustion, and physical and cognitive fatigue (15). Among health care workers burnout syndrome has been directly linked to the onset of depression, nervousness, helplessness, and anxiety (12, 16).

The COVID-19 pandemic has caused a global imbalance in negative versus positive emotions across all populations, leading to burnout across the entire healthcare system (5). The current study examines whether healthcare workers’ work environment affects their psychological distress, COVID-related concerns, and burnout levels. Healthcare workers working in the underground hospital were compared to a similar population who did not work in the underground hospital at that time. Additionally, taking into account that the pandemic is yet to be resolved, we examined the impact of working in an underground facility and other personality- and profession-related factors on burnout to better understand how to optimally manage similar situations in the future.

## Methods

### Participants

The survey included healthcare medical and paramedical staff who worked in the underground hospital during the peak of the COVID-19 pandemic for a minimum of 2 weeks and healthcare staff who worked in their regular environment (control group). The study was approved by the Rambam Helsinki committee and by the IRB (Institutional Review Board) of the Faculty of Education, University of Haifa.

#### Non-underground sample

Data of control participants were taken from (17). This group included 40 healthcare workers from northern Israel who did not work at the underground hospital. They filled out the same questionnaire as the workers in the underground sample, except for the questions related to the work in the underground hospital in the “COVID-19 Concerns Questionnaire.” The control group received the online survey in April 2020, approximately half a year before the underground group filled in the survey.

#### Underground sample

This group included 76 participants. Initial contact was established with the staff of the underground hospital during February–June, 2021, during which an initial selection was made according to the study’s inclusion criteria.

#### Inclusion criteria

(1) Hospital employees over the age of 18. (2) Employees who had been working or previously worked in the COVID-19 wards in the underground hospital for a minimum continuous period of 2 weeks.

#### Exclusion criteria

(1) Minors. (2) Previous history of mental illness.

### Procedure

After signing an informed consent form, participants received a text message with an attached link to an Online questionnaire (Qualtrics, Online Survey Questionnaire). This was sent only once and in Hebrew. Filling out the questionnaire took about 20 min.

#### Questionnaires

The online survey included six questionnaires:

**Demographic Questionnaire**. This questionnaire included questions about gender, age, origin, professional position and placement, salary and previous work experience. It also included questions related to the pandemic itself, for example: “Have you been previously diagnosed with a COVID-19 infection?”

**COVID-19 concerns questionnaire** [**based on** (17)]. This questionnaire assessed the level of stress experienced by the employee due to the pandemic and, particularly, due to work in the underground hospital (e.g., the fear of becoming infected, of infecting family members, and fear of working with COVID-19 infected patients). This questionnaire also included questions about concerns related to the employee’s personal socioeconomic situation, personal appearance and relationships during the pandemic. Participants responded on a 5-point scale ranging from 1 (not at all) to 5 (very much). This questionnaire was based on a questionnaires developed during the SARS outbreak (18).

**The Depression Anxiety Stress Scale (DASS)** (19, 20). The DASS is a 21-item self-report instrument designed to measure the three related negative emotional states depression, anxiety and stress. Participants were asked to rate how often they experienced each item in the past week on a four-point scale ranging from 0 (does not apply to me at all) to 3 (applies to me very much or most of the time).

**The Penn State Worry Questionnaire (PSWQ)** (21). The PSWQ is a 16-item questionnaire assessing personality traits, such as the tendency to worry and the severity of concern. Participants were instructed to indicate the degree to which they regarded each item as typical of them on a five-point scale ranging from 1 (not at all typical of me) to 5 (very typical of me).

**Emotion Regulation Questionnaire (ERQ)** (22). The ERQ consists of 10 statements, examining two emotion regulation strategies; reappraisal and suppression. Participants were asked to rate whether they agreed or disagreed with each statement on a scale of 1 to 7 (1 = strongly disagree, 7 = strongly agree).

**Shirom - Melamed Burnout Measure (SMBM)** (15). The SMBM assesses burnout at the workplace. It consists of 16 items divided into three sub-scales: physical fatigue (six items), cognitive weariness (six items) and emotional exhaustion (four items). Participants were asked to answer on a seven-point scale ranging from 1 = “almost never” to 5 = “almost always.”

### Analytical strategy

Statistical significance was set *a priori* to <0.05 (two-tailed), with all analyses conducted using IBM SPSS Statistics version 26 (IBM, Armonk, NY). We first performed independent-samples *t*-tests to examine differences between the two study groups. Then, a hierarchical linear regression analysis was performed to determine the predictors of job burnout. In the first step, the demographic characteristics of group (underground hospital employees vs. health care workers in general), profession type (physician vs. non-physician), age and gender were entered into the model. In the second step, situational factors related to distress, such as overall psychological distress (total DASS score) and COVID-19 concerns were added. In the third step, trait characteristics related to emotion regulation tendencies (reappraisal, suppression, and worry) and personality traits were added to the model.

## Results

A total of 116 healthcare workers participated in the study, 76 employees who worked in the underground hospital versus 40 employees who did not (control group). 68 of the participants (58.6%) were women, mean age was 34.1 (SD = 8.04). Full demographic information on all participants in the study is given in Table 1.

**Table 1:** Demographic characteristics of the study sample.

		Frequency	Percentage
Occupation	Physician	49	42.2	Nurse	49	42.2	Other	18	15.6
Work experience	Under 1 Year	15	12.9	1–5 Years	49	42.2	5–10 Years	31	26.7	10–15 Years	6	5.2	15–20 Years	5	4.3	Over 20 Years	10	8.6
Job percentage	Under 50%	9	7.8	50%	5	4.3	75–88%	10	8.6	100%	92	79.3
Gender	Male	48	41.4	Female	68	58.6
Marital status	Single	33	28.4	Long-Term Relationship	10	8.6	Married	69	59.5	Divorced	4	3.4
No. children	0	55	47.4	1	18	15.5	2	20	17.2	3	19	16.4	4	2	1.7	5	1	0.9
Religion	Jewish	44	37.9	Christian	35	30.2	Muslim	31	26.7	Druze	1	0.9	Other/Atheist	5	4.3
Religious level	Orthodox	17	14.7	Moderate	34	29.3	Secular	62	53.4	Other	3	2.6
SES	Very High	4	3.4	Higher than Average	59	50.9	Average	41	35.3	Lower than Average	12	10.3
COVID_test	Yes	72	62.1	No	44	37.9
COVID-19 infection	Yes	8	6.9	No	68	58.6	N/A	40	34.5

### Group differences in psychological distress and burnout

We conducted five independent-samples *t*-tests to examine differences between the two groups in DASS total and sub-scale scores, SMBM scores and COVID-19 concerns. No significant differences between Rambam underground hospital workers and the control group were found in the DASS total scores (*M* = 35.94, *SD* = 14.3 and *M* = 36.07, *SD* = 12.27, respectively), *t*_(114)_ = −0.046, *ns*; DASS depression scores (*M* = 11.94, *SD* = 5.16 and *M* = 11.87, *SD* = 4.38, respectively), *t*_(114)_ = 0.075, *ns*; DASS anxiety scores (*M* = 10.06, *SD* = 4.42 and *M* = 9.9, *SD* = 3.88, respectively), *t*_(114)_ = 0.2, *ns*; and DASS stress scores (*M* = 13.93, *SD* = 5.99 and *M* = 14.29, *SD* = 5.26, respectively), *t*_(114)_ = −0.321, *ns*. SMBM scores also showed no significant difference between Rambam underground hospital workers (*M* = 3.39, *SD* = 1.2) and the control group (*M* = 3.21, *SD* = 1.03), *t*_(114)_ = 0. 822, *ns*.

Surprisingly, COVID-19 concern scores showed a significant difference, with lower levels of concerns among Rambam hospital workers (*M* = 2.9, *SD* = 0.73) than other healthcare workers (*M* = 3.47, *SD* = 0.76, *t*_(114)_ = −3.974, *p* < 0.001). Results are given in Table 2.

**Table 2:** Differences in DASS, SMBM, and COVID-19 concerns (Mean_all_concerns) scores between the two groups.

Location						
Scales	Rambam underground hospital workers (*n* = 76)		Healthcare workers in general (control group) (*n* = 40)			
	*M*	*SD*	*M*	*SD*	*t*_(114)_	*p*
DASS – total	35.94	14.3	36.07	12.27	−0.046	0.96
DASS – depression	11.94	5.16	11.87	4.38	0.075	0.94
Dass – anxiety	10.06	4.42	9.9	3.88	0.2	0.84
Dass – stress	13.93	5.99	14.29	5.26	−0.321	0.75
SMBM	3.39	1.2	3.21	1.03	0.822	0.41
Mean_all_concerns	2.9	0.73	3.47	0.76	−3.974	<0.001

**Predicting job burnout.** Hierarchical linear regression analysis was performed to identify the significant predictors of job burnout among healthcare workers (Tables 3, 4) with burnout scores as dependent variable. The first step, which included demographic characteristics of age, gender, group (underground workers vs. control group) and profession (physician vs. non-physician), accounted for significant variance in burnout, *R^2^* = 0.093, *F*(4,111) = 2.8, *p* = 0.028. In this step participants’ profession (physician) significantly predicted burnout (*β* = 0.271, *p* = 0.007). As mentioned above, group (variable) did not predict burnout (*p* = 0.322).

**Table 3:** Predictors of job burnout.

								95.0% Confidence interval for B	
	Predictors	R2	B	*S.E.*	*β*	*t*	*p*	Lower bound	Upper bound
Step 1	Group	0.093	0.233	0.235	0.097	0.995	0.322	−0.231	0.698	Age	0.008	0.014	0.056	0.594	0.553	−0.019	0.035	Doctor	0.626	0.228	0.271	2.745	0.007	0.174	1.078	Gender_level	0.507	0.228	0.218	2.223	0.028	0.055	0.958
Step 2	Group	0.484	0.185	0.190	0.077	0.974	0.332	−0.192	0.563	Age	0.015	0.010	0.106	1.455	0.149	−0.005	0.036	Doctor	0.770	0.176	0.333	4.377	0.000	0.421	1.118	Gender_level	0.003	0.182	0.001	0.016	0.987	−0.358	0.364	DASS_total	0.048	0.007	0.563	7.004	0.000	0.034	0.061
	Mean_all_concerns		0.273	0.123	0.188	2.221	0.028	0.029	0.517
Step 3	Group	0.516	0.113	0.189	0.047	0.598	0.551	−0.261	0.488	Age	0.017	0.010	0.116	1.608	0.111	−0.004	0.037	Doctor	0.754	0.175	0.326	4.300	0.000	0.406	1.101	Gender_level	−0.091	0.199	−0.039	−0.457	0.649	−0.485	0.303	DASS_total	0.041	0.007	0.491	5.836	0.000	0.027	0.056	Mean_all_concerns	0.215	0.126	0.148	1.709	0.090	−0.034	0.464	PSWQ_total	0.019	0.008	0.188	2.308	0.023	0.003	0.035	ERQ_reappraisal	0.000	0.011	0.002	0.028	0.977	−0.021	0.021	ERQ_suppression	0.016	0.017	0.076	0.980	0.330	−0.017	0.050

**Table 4:** Predictors of job burnout, model summary.

Model summary									
Model	R	R square	Adjusted R square	Std. error of the estimate	Change statistics				
					R square change	F change	df1	df2	Sig. F change
1	0.305^a^	0.093	0.060	1.11222	0.093	2.840	4	111	0.028
2	0.696^b^	0.484	0.455	0.84669	0.391	41.270	2	109	0.000
3	0.718^c^	0.516	0.475	0.83118	0.032	2.368	3	106	0.075

a Predictors: (Constant), gender_level, age, group, doctor.

b Predictors: (Constant), gender_level, age, group, doctor, DASS_total, mean_all_concerns.

c Predictors: (Constant), gender_level, age, group, doctor, DASS_total, mean_all_concerns, ERQ_reappraisal, ERQ_suppression, PSWQ_total.

The second step, in which DASS total and COVID-19 concerns were added, was significant (2,109 = 41.2, *p* < 0.001) and accounted for 48% of the variance in burnout. In this step, psychological distress (total DASS score) significantly predicted burnout, (*β* = 0.56, *t* = 7.004, *p* < 0.001), similar to COVID-19 concerns (*β* = 0.188, *t* = 2.221, *p* = 0.028) and profession (physician vs. non physician) (*β* = 0.333, *t* = 4.37, *p* < 0.001).

The third step included emotion regulation tendencies, reappraisal, suppression, and worry. In this model worry significantly predicted burnout (*β* = 0.188, *t* = 2.3, *p* = 0.023), similar to profession (*β* = 0.326, *t* = 4.3, *p* < 0.001) and psychological distress (*β* = 0.491, *t* = 5.836, *p < 0.001*). Note that concerns about COVID-19 did not reach significance in this step (*β* = 0.148, *t* = 1.7, *p* = 0.09).

## Discussion

The COVID-19 crisis forced major changes in healthcare facilities including opening new sites. Our study was conducted in northern Israel during 2021, half a year after transforming the Sammy Ofer fortified underground hospital into a COVID-19 treatment center. At that time several studies from Israel and around the world showed significant correlation between psychological distress and burnout among healthcare workers in the face of the COVID-19 pandemic (15, 23–28, 43). These studies led us to examine the effects on healthcare workers of working in an underground facility and to examine the factors predicting job burnout.

Based on the concept of “environmental medical syndromes” (29), we predicted that working in the underground hospital in sub-optimal conditions (no windows and view of the outside world, no sunlight, closed-loop air circulation, wearing restrictive protective equipment, longer shifts) would cause significantly greater psychological distress, COVID-19 concerns and burnout than in healthcare workers not exposed to the same work conditions.

Surprisingly, our results did not support our hypotheses; there was no significant differences in psychological distress (Total-DASS) nor in burnout between the two groups. The study did, however, reveal a significant difference in COVID-19 concerns between the two groups, with lower mean scores among those working in the underground hospital. One explanation for this result may be the small sample size, particularly of the control group. Another cause may be that Rambam employees filled in the questionnaires approximately half a year later than the control group, when the expression of the COVID-19 pandemic was clearer and less acute and the benefits of protective gear were better understood. Also, Rambam Hospital employed social and psychological support teams for employees, which may have helped lower their distress.

We examined factors that may contribute to developing burnout. Our findings suggest that being a physician, having an intrinsic trait of being overly worried and experiencing psychological distress were significant predictors of job burnout among healthcare workers generally, whereas group (underground vs. controls) did not predict job burnout. Our results agree with previous findings around the world of a correlation between being a physicians and job burnout in the everyday work environment, regardless of the COVID-19 pandemic (30–34).

We hypothesized that physicians, who were exposed to COVID-19 patients, would be more likely to develop burnout syndrome (2). Due to the lack of a comparative follow-up questionnaires at different periods, testing this hypothesis requires more research.

Although several studies have investigated the link between psychological variables and job burnout (3, 10, 21, 33, 35–38), there are almost no studies on the correlation between the personality trait tendency to worry with job burnout among healthcare workers. We found that worrying greatly did predict job burnout independently of profession and external circumstances. Our findings are in line with previous studies demonstrating positive correlations between psychological distress and burnout during regular work (36, 39, 40), as well as during a pandemic (15, 27, 41). Here, psychological distress predicted burnout to a greater degree than worry (*β* = 0.491 vs. *β* = 0.188).

Previous studies identified moderate to severe levels of psychological distress and burnout among healthcare workers during the COVID 19 pandemic (15, 24–26, 28, 38). However, to the best of our knowledge, no studies have examined the direct effect of COVID-19 concerns on job burnout among healthcare workers. Our study suggests a marginally significant association between COVID-19 concerns and job burnout among healthcare workers, making it a valuable addition to the available data.

The current study has several limitations. First, the underground sample was much larger than the control group and both groups consisted predominantly of women. Thus, the results may be less generalizable. Second, the current study was cross-sectional, which does not allow examination of directionality between variables. Furthermore, the two groups filled out the questionnaire half a year apart within different COVID-19 waves.

## Data availability statement

The original contributions presented in the study are included in the article/supplementary material, further inquiries can be directed to the corresponding author/s.

## Ethics statement

The studies involving human participants were reviewed and approved by Rambam Helsinki committee and by the IRB (Institutional Review Board) of the Faculty of Education, University of Haifa. The patients/participants provided their written informed consent to participate in this study.

## Author contributions

NC, MK, and LN contributed to conception and design of the study. IM and LN organized the database. TL performed the statistical analysis. LN wrote the first draft of the manuscript. All authors contributed to the article and approved the submitted version.

## Conflict of interest

The authors declare that the research was conducted in the absence of any commercial or financial relationships that could be construed as a potential conflict of interest.

## Publisher’s note

All claims expressed in this article are solely those of the authors and do not necessarily represent those of their affiliated organizations, or those of the publisher, the editors and the reviewers. Any product that may be evaluated in this article, or claim that may be made by its manufacturer, is not guaranteed or endorsed by the publisher.
